# Fish and Seafood Safety: Human Exposure to Toxic Metals from the Aquatic Environment and Fish in Central Asia

**DOI:** 10.3390/ijms25031590

**Published:** 2024-01-27

**Authors:** Gulnur Zhuzzhassarova, Faranak Azarbayjani, Galia Zamaratskaia

**Affiliations:** 1 Department of Veterinary Sanitation, S. Seifullin Kazakh Agro-Technical University, Astana 010 011, Kazakhstan; gulnur900607@gmail.com; 2 Department of Pharmaceutical Biosciences, Uppsala University, 751 24 Uppsala, Sweden; faranak.azarbayjani@uu.se; 3 Department of Molecular Sciences, Swedish University of Agricultural Sciences, 750 07 Uppsala, Sweden; 4 South Bohemian Research Center of Aquaculture and Biodiversity of Hydrocenoses, Faculty of Fisheries and Protection of Waters, University of South Bohemia in Ceske Budejovice, Zatisi 728/II, 389 25 Vodnany, Czech Republic

**Keywords:** fish, toxic metals, arsenic, mercury, cadmium, lead

## Abstract

Toxic metals that are released into aquatic environments from natural and anthropogenic sources are absorbed by aquatic organisms and may threaten the health of both aquatic organisms and humans. Despite this, there have been limited studies on the metal concentrations in fish and humans in Central Asia. This study summarizes the presence of the toxic metals arsenic (As), mercury (Hg), cadmium (Cd), and lead (Pb) in aquatic bodies, fish, and seafood products and conducts a risk assessment. While certain areas show a notable increase in fish and seafood consumption, the overall intake in Central Asia remains below recommended levels. However, in regions with high fish consumption, there is a potential for elevated exposure to toxic metals, especially Hg. The risk of exposure to toxic metals in fish and seafood in Central Asia emerges as a significant concern. Comprehensive monitoring, regulation, and remediation efforts are imperative to ensure the safety of water sources and food consumption in the region. Public awareness campaigns and the establishment of dietary guidelines play a crucial role in minimizing the health risks associated with consumption.

## 1. Introduction

Fish represents a valuable source of protein and lipids in the human diet, particularly the long-chain-3 polyunsaturated fatty acids docosahexaenoic acid (DHA) and eicosapentaenoic acid (EPA). These fatty acids are incorporated into the phospholipids of cellular membranes and are essential for normal heart, cardiovascular, and brain functions, can reduce the risk of coronary heart diseases, and play a beneficial role in inflammatory conditions [1]. DHA and EPA are also crucial during development. Nutrition guidelines recommend increasing fish consumption to two to three times per week, especially “fatty” species that are high in DHA and EPA, such as salmon, mackerel, sardines, and trout. Another omega-3 fatty acid, alpha-linolenic acid, can be converted into EPA and DHA but this conversion is fairly limited in humans. This makes fish and seafood an exclusive source of EPA and DHA. However, only a small portion of the population in Central Asia consumes fish in accordance with the nutritional recommendations [2,3,4]. Interestingly, the European Society of Cardiology has classified Kazakhstan as a high-risk country for the development of cardiovascular diseases and Kyrgyzstan and Uzbekistan as very high-risk countries [5]. This classification does not include Tajikistan and Turkmenistan.

Public awareness regarding dietary exposure to environmental contaminants, which can be accumulated in fish, is growing and compromises adequate fish intake. Indeed, the accumulation of toxic compounds in the human body can have severe health consequences including developmental abnormalities, neuromuscular defects, mental illness, kidney diseases, and cancer [6]. Toxic compounds released into aquatic environments from natural and anthropogenic sources are absorbed by aquatic organisms. Even low toxic metal levels may threaten the health of aquatic organisms and humans [7]. Previous studies have detected high concentrations of toxic metals in water and fish in rivers and seas in Central Asia [7,8,9,10,11].

The toxic metals arsenic (As), mercury (Hg), cadmium (Cd), and lead (Pb) are the most common heavy metals that induce human poisoning. Fish and aquatic product consumption is the major pathway for human exposure to Hg and As and, to a lesser extent, Cd and Pb [12]. These compounds are persistent and not biodegradable. Human industrial activity is the primary source of the inorganic Hg released and eventually deposited in aquatic environments. Microorganisms in aquatic environments, like sulfate-reducing bacteria, perform biomethylation of Hg to produce methyl-Hg. Methyl-Hg is taken up by phytoplankton, which is then consumed by fish. Methyl-Hg bioaccumulates and biomagnifies. Thus, large predatory fish are more likely to have high levels of Hg as a result of eating many smaller fish that have accumulated Hg through phytoplankton ingestion [12]. Then, methyl-Hg eventually enters the human body through the consumption of fish and seafood [7,12]. The metalloid As is also widespread in the aquatic environment due to natural and anthropogenic processes. Fish are continuously exposed to it through their gills and skin. The exposure of freshwater fish to As results in its bioaccumulation, mainly in liver and kidney tissues. Cd and Pb have been widely recognized as pollutants in waters worldwide and as a threat to aquatic organisms [7]. However, cereals and vegetables are considered the primary sources of dietary Cd and Pb. Additionally, Pb concentrations might be high in game meat shot with lead ammunition.

Numerous studies have reported the levels of toxic metals in aquatic environments and food products worldwide [13,14,15]. In Central Asia, very few studies have been performed on toxic metal contamination of aquatic food products, with no well-documented human risk assessments. Central Asia includes the following countries: Kazakhstan, Kyrgyzstan, Tajikistan, Turkmenistan, and Uzbekistan. These countries are regularly ranked among the most polluted air in the world [16], have experienced water stress, and suffer from contamination with toxic metals [17,18]. Many studies have documented an increased risk of respiratory and cardiovascular diseases and a higher overall mortality rate in the polluted regions of Central Asia [19,20]. Toxic metal contamination in aquatic environments is mainly due to natural processes and anthropogenic activities. The industrial sector is an important part of the economic development of Central Asia and is the largest anthropogenic source of aquatic contamination with heavy metals. Being a part of the former Soviet Union, Central Asia experienced enormous developments of mining and chemical production, which resulted in the release of high amounts of contaminants into the environment, including toxic metals [21]. Additionally, Soviet nuclear weapons testing in some areas contributed to the contamination. The nuclear weapons testing can also lead to the release of radioactive isotopes and toxic metals into the environment, affecting soil, water, and air quality. The actual level of co-contamination would depend on various factors, such as the proximity of the testing sites, the type and scale of testing conducted, and local environmental conditions. Toxic metals are potentially accumulated in sediments, water, and aquatic organisms and transferred to humans through the food chain.

Several studies have reported elevated levels of toxic metals in the blood and hair of residents of Central Asia [22,23].

This review aims to evaluate and summarize the presence of selected toxic metals, such as Hg, As, Cd, and Pb in aquatic environments and aquatic products in Central Asia and conduct a risk assessment based on the average per capita intake of fish and seafood products. The authors independently searched for scientific articles and reports without language restrictions. The databases that were searched were PubMed, Web of Science, and Scopus databases. First, each toxic metal was individually searched for its levels in waters and foods in Central Asia. Then, the metals were searched per country or city name. As some combinations of search words have few publications, Google Scholar databases were used to allow a broader search. In Google Scholar, keywords were used in English, Russian, and Central Asian languages.

## 2. Toxic Metals

Arsenic (As) is a metalloid that is ubiquitously present in the environment. Inorganic As is toxic to humans and can lead to skin lesions, peripheral neuropathy, gastrointestinal disorders, and cancer of the skin and internal organs [24,25]. Organic As compounds are less harmful and are rapidly eliminated by the body. As compounds can be released into the aquatic environment from both natural and anthropogenic sources [26]. In fish and seafood, As is primarily present in the organic form, with arsenobetaine being the major compound [27].

Mercury (Hg) is one of the most widespread contaminants in the aquatic environment. Organic Hg is considered the most hazardous form of Hg exposure. Neurotoxicity is a major concern with methylmercury (MeHg) exposure, especially for the developing nervous system [28]. Hg can bioaccumulate and its concentration increases in the food chain, resulting in higher concentrations of Hg in predatory fish [29,30].

Cadmium (Cd) is found in the environment naturally and as a result of industrial and agricultural activities. Long-term exposure to Cd can lead to renal dysfunction, osteoporosis, and some forms of cancer [31,32]. High Cd concentrations have been detected in shellfish but not in fish [33,34].

Lead (Pb) is a naturally occurring toxic metal found in the Earth’s crust. Human poisoning was often associated with Pb-containing gasoline and this gasoline was gradually phased out in many countries during the 1990s. Pb can adversely affect the central nervous system in developing infants [35]. In adults, Pb may also affect blood pressure, reproductive and kidney functions, and cause mutagenesis. The Pb content in fish is generally low but can be high in cephalopod mollusks [36,37].

## 3. Distribution of Toxic Metals in the Water of Rivers, Lakes, and Seas in Central Asia

Central Asia, covering approximately 2.72 million km^2^ of dryland, faces significant water challenges, particularly within its extensive endorheic basins. These basins, devoid of direct connections to the ocean, suffer from constrained surface and groundwater resources. Over the past century, intensified demands from agriculture, energy, and a growing population have resulted in detrimental environmental consequences, including diverse pollution in rivers, lakes, and groundwater. This degradation threatens both aquatic ecosystems and the socioeconomic development of the region [17,38,39]. In the lowlands of Central Asia, major river systems and lake basins, such as the Amu Darya-Syr Darya-Aral Sea Basin and the Ili River-Lake Balkhash Basin, grapple with substantial changes [39]. Natural ecosystems, originally adapted to the region’s natural water availability, have undergone significant disruptions due to population growth and intensified water-consuming economic activities. This shift has altered socioecological systems, posing a high probability of water-induced changes with potentially severe impacts on human societies.

The environmental contamination of water and sediments in Central Asia is becoming increasingly prominent. The Amu Darya Basin was found to be at a moderate pollution level of toxic metals, as assessed using several pollution indices and the ecological risk index [11]. According to Zhan et al. [11], toxic metal contaminations in the surface sediments of the lower Amu Darya Basin were assessed using pollution indices. Two key pollution indices, the single-factor pollution index (PI) and pollution load index (PLI), were utilized to evaluate contamination levels. The PI gauges individual toxic metal contamination, while the PLI combines these values for an overall pollution assessment. Concentrations of various toxic metals were measured, revealing a descending order: Zn > Cr > Ni > Cu > Pb > Co > Cd. Spatially, heavier metal accumulation was noted in cities and agricultural areas. Source identification methods, including correlation analysis and the positive matrix factorization (PMF) model, identified four distinct sources of contamination: natural sources, industrial discharge, agricultural sources, and a mixed source of traffic and mining activities. Geochemical background values (GBVs) were compared to regional values for context.

The spatial distribution maps revealed notable enrichment of Cr, Ni, and Cu in irrigated agricultural areas, while Zn, Pb, and Cd were predominantly concentrated in urban zones. Identified sources of these metals included natural factors, industrial discharges, agricultural activities, and a combination of traffic and mining, contributing 33.5%, 11.4%, 34.2%, and 20.9% to the overall contamination, respectively. The GBVs for Cd, Zn, Pb, Cu, Ni, Cr, and Co in the Amu Daryas Basin were measured at 0.27, 58.9, 14.6, 20.3, 25.8, 53.4, and 9.80 mg/kg, respectively, aligning closely with regional background values from lake sediments. Contamination with Zn, Cr, Ni, Cu, and Co are outside the scope of this study. In summary, these pollution levels are enough to impact aquatic ecosystems, raising health concerns for the local population.

Despite this, high levels of toxic metals and other contaminants were detected in the blood of children and pregnant women in the lower Amu Darya Basin, leading to several health problems, including high infant mortality, low birth weight, growth retardation, acute respiratory diseases, anemia, miscarriage, and other recorded disorders [40,41,42,43].

Rzymski et al. [9] investigated toxic metal contamination in the lower course of the Syr Darya River and the small Aral Sea. They reported that in the Syr Darya River, As was detected at the level of 35.8 ± 21.2 (mean ± standard deviation). Overall, the measured As levels in Syr Darya and the Small Aral Sea were 2–7 times higher than the WHO guideline level of 10 μg/L [44]. The concentrations of Hg were below detection limits in both the Syr Darya River and the small Aral Sea region and thus below the WHO guideline level for Hg (6.0 μg/L) [44]. Cd and Pb were detected in the Syr Darya river and, at some locations, the Cd and Pb concentrations [9,45,46] exceeded the WHO guideline levels (3.0 and 10 μg/L, respectively) [44]. According to previous Kazakhstan Ministry of Ecology, Geology, and Natural Resources reports, the Syr Darya water in the Turkestan and Kyzylorda regions is only suitable for irrigation and industrial uses [47].

In the Ili River (Kazakhstan), the levels of Cd varied from 1.7 to 28.7 and the levels of Pb, from 0.2 to 87.0 μg/L, exceeded the WHO guideline levels more than five times at some locations [10]. Relatively high levels of As (approximately 16 μg/L) were measured in Issyk-Kul Lake (Kyrgyzstan) [48], exceeding the accepted drinking water level. The levels of Pb in Issyk-Kul Lake were approximately 0.09 µg/L [48].

The contamination of rivers and reservoirs with toxic metals varies depending on location and season. During flood periods, the sediments release Hg, leading to increased Hg concentrations in the water [8]. In the river Nura, the highest concentrations of Hg were observed during spring and summer seasons followed by autumn and winter [49]. In contrast, seasonal variations in Hg concentrations in the Syr Darya River was low, with a low percentage of samples (0.85%) exceeding the guideline levels. The highest concentration of Cd in the Syr Darya River was observed in winter but Cd concentrations exceeded the guideline levels in all seasons [49]. The variations in As concentrations in the river Nura were also low, whereas As in the river Ili in winter and spring samples exceeded the WHO guidelines [49].

The Karbide chemical plant is located on the river Nura in northeast–central Kazakhstan and the uncontrolled release of wastewater from the plant has resulted in serious contamination of the river [8]. This area, particularly Temirtau City, is known for high contamination with mercury and the citizens of the area were reported to have a higher risk of hypertension and higher blood concentrations of inflammatory markers [50]. Heaven et al. [8] reported a range of Hg concentrations of 0.46–5.36 μg/L in the river Nura. Shinetova et al. [50] measured 4.5 ± 9.8 μg/L (mean ± standard deviation) of Hg in highly contaminated parts and 0.03 ± 0.08 μg/L in low contaminated parts of the river Nura.

Springs in Central Asia are traditionally popular as tourist attractions and are widely used for balneological and religious purposes and as a source of drinking water. For example, western Kazakhstan attracts pilgrims with many sacred historical constructions and natural springs, which are considered to have healing properties. In balneotherapy, people immerse themselves in spring water for health benefits or apply water to the skin for therapeutic purposes. In religious practices, people might use spring water for ritual cleansing. Exposure to toxic metals via skin in both cases would depend on the composition of the spring water. Regular analysis of the spring water would be necessary to assess the risk of exposure. Monitoring studies of the spring waters in the Aktobe region, western Kazakhstan did not detect the presence of Pb but identified three springs, where Cd concentrations exceeded the WHO guideline level as follows: in Islambulak by three times and in Bulak ayly and Akshat by five times [51]. In the Atyrau region, neither Cd nor Pb exceeded the guideline levels [52].

The presence of toxic metals in the aquatic environment poses substantial health risks to fish and humans. Thus, careful monitoring and human health risk assessments should be routinely performed.

## 4. Occurrence of Toxic Metals in Fish and Seafood in Central Asia

Fish products represent one of the main uptake routes of toxic metals by humans, especially MeHg. Fish and meat also contain Cd and As, making them potential sources of dietary exposure. Until recently, contamination with toxic metals and their food sources has been largely ignored in Central Asia. However, monitoring of the accumulated toxic metals in fish tissues is essential for evaluation of pollution and environmental hazards. Nowadays, concerns over the quality of food and its health effects are growing. Dietary intakes of toxic metals should be maintained within the permissible limits. To minimize human exposure to toxic metals, the Eurasian Customs Union established maximum permissible concentrations of toxic metals in fish and seafood products (on food safety) (Table 1). MeHg comprises the largest portion (up to 100%) of the total mercury in seafood. The concentration of MeHg in fish depends on the species, feed, size, and age. Large predatory fish are known to contain the highest concentrations of MeHg.

In 2007 and 2008, the results of the collaboration between the Norway, Kazakhstan, Kyrgyzstan, and Tajikistan (JNKKT) project and the NATO SfP RESCA project demonstrated that overall, the muscle Hg concentrations in studied fish species were low and did not represent any health risk [48,54]. In those projects, the following fish were sampled at the following locations: (1) Kurday, Kazakhstan; (2) Kadji Sai, situated on the southern coast of Issyk-Kul Lake, Kyrgyzstan; and (3) Taboshar and Digmai, Tajikistan. These locations were selected based on the fact that during the 1950s and 1960s, uranium mining sites operated as part of the USSR nuclear weapon program and large amounts of uranium-tailing materials and toxic compounds are still found at the sites, close to residential areas.

Later studies on fish and seafood in Central Asia generally reported levels of toxic metals below maximum permissible concentrations, although the levels greatly varied depending on the sampling location and fish species (Table 2). However, at some locations, fish with high Hg concentrations were detected. Lake Balkyldak in North Kazakhstan is a highly contaminated area because of the chlor-alkali plant in Pavlodar, which operated during 1975–1993 and was based on Hg-cell technology. In 2001–2002, fish caught from Lake Balkyldak had high Hg levels (0.16–2.2 mg/kg) [55] and in 2006–2007, the Hg levels were slightly lower (1–1.5 mg/kg) [56]. Nevertheless, Hg levels in the majority of fish exceeded the maximum permissible concentration of Hg in freshwater fish in Kazakhstan of 0.3 mg/kg [55,56]. Moreover, Kaliyeva and Ermienko [57] observed several physiological, biochemical, and morphological abnormalities in fish from Lake Balkyldak.

The poor environmental conditions in the Pavlodar region are also coupled with the former activity of the Semipalatinsk Nuclear Test Site, the test site for testing nuclear weapons during the Soviet era [63]. The Semipalatinsk nuclear test site covers 18,500 km^2^ in the northeast of Kazakhstan near the city of Semey. In 1949–1989, the Soviet Union intensively conducted nuclear tests there but the health consequences of exposure to radiation, toxic metals, and other contamination are still unclear. A large number of studies have focused on assessing the radioactive contamination due to the nuclear tests at the Semipalatinsk nuclear test site. However, more data are needed on the contamination with toxic metals. Additionally, co-exposure effects should be considered. Studies of this kind are scarce. Sharov et al. [21] analyzed toxic hotspots in eight former Soviet countries using a global contaminated sites database. The research has identified 424 polluted sites in Armenia, Azerbaijan, Kazakhstan, Kyrgyzstan, Russia, Tajikistan, Ukraine, and Uzbekistan, assessing contamination levels of seven key pollutants (pesticides, lead, radioactive metals, arsenic, mercury, chromium, and cadmium). These sites collectively endanger an estimated 6.2 million residents, with existing data likely capturing only a fraction of actual contaminated sites, indicating potentially severe public health consequences. The study emphasizes the need for additional assessments to comprehensively understand the risks posed by toxic pollution in the region.

The levels of Hg in fish (roach and perch) from the Intumak Reservoir, which is located on the Nura River, also exceeded the permissible level [64]. The concentrations of Pb in roach muscle tissue from the Shardara Reservoir, situated in the southeastern part of the Kyzylkum desert, along the river Syr Darya, ranged from 0.1 to 3.8 μg/g wet weight [45].

Integrated water management approaches like water resources management and the water–food–energy nexus are crucial to securing a sustainable future. This thematic issue delves into the intricate water challenges of Central Asia, offering insights derived from hydrological research, water quality investigations, and ecosystem assessments. Within this framework, reviews and case studies provide field-tested solutions for effectively managing the region’s water resources [38,39,65].

## 5. Intake of Fish and Seafood in Central Asia

**Figure 1:**
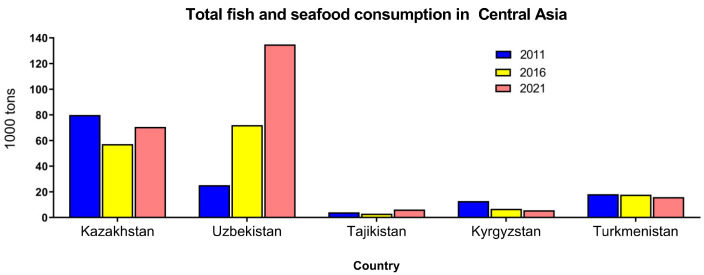
Total fish and seafood consumption in Central Asia per country from 2011 to 2021. Data were retrieved from FAOSTAT [4].

**Figure 2:**
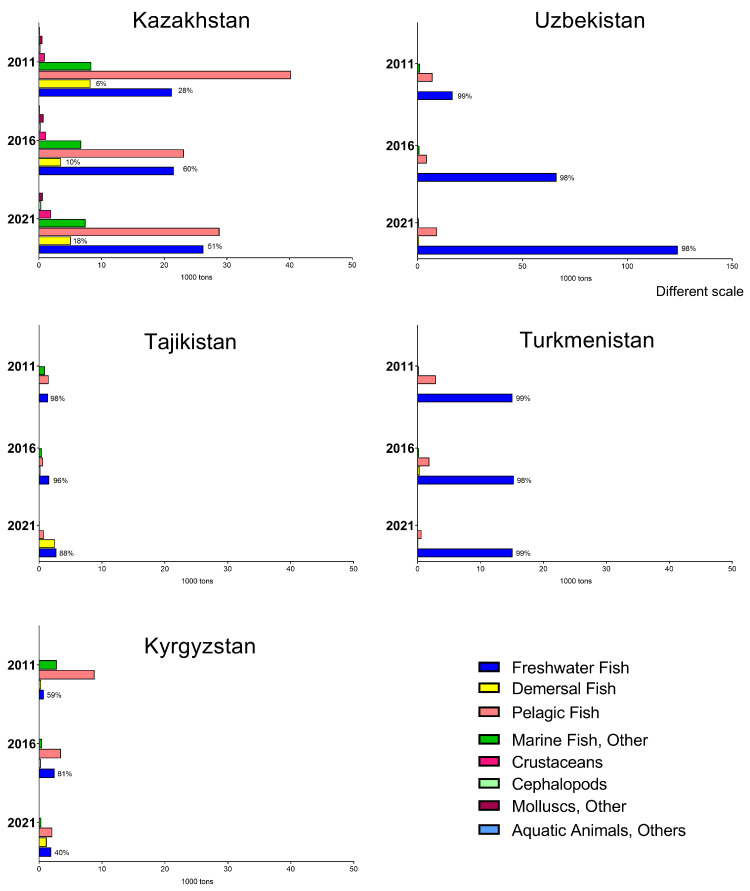
Fish and seafood consumption per food group in Central Asia per country in 2011, 2016, and 2021. Data were retrieved from FAOSTAT [4]. Numbers on the bars indicate the domestic supply quantity (%). If no numbers are provided, the consumption was based only on imported products.

Achieving food sufficiency to meet dietary recommendations is one of the major objectives of the agricultural sector. However, current research on fish and seafood consumption in Central Asia is limited. According to FAOSTAT data, there are substantial variations in fish and seafood consumption among Central Asian countries [4] (Figure 1). These differences might be related to the different levels of supply of fish and seafood, income levels, awareness of healthy diets, gastronomic traditions, and consumer preferences. During the last 20 years, a significant increase in fish and seafood consumption was observed in Uzbekistan possibly due to increased supplies and changed consumer preferences. A slight increase was also recorded in Tajikistan, while consumption somewhat decreased in Kazakhstan, Turkmenistan, and Kyrgyzstan (Figure 1). Large variations between countries were observed in the most frequently consumed fish species and seafood (Figure 2). In Kazakhstan and Kyrgyzstan, the most consumed fish group was pelagic fish followed by freshwater fish. In contrast, Uzbekistan, Tajikistan, and Turkmenistan mainly consume freshwater fish and, to a lesser extent, other fish groups. Interestingly, the consumption of crustaceans, mollusks, and other aquatic food items was negligible in Uzbekistan, Tajikistan, Kyrgyzstan, and Turkmenistan (Figure 2). It should be emphasized that this consumption was calculated for the entire population within each country and does not reflect the per capita consumption. The estimated per capita consumption of fish and seafood in Central Asia is presented in Table 3. There was a huge gap between the country with the lowest consumption (0.63 kg/year in Tajikistan) and the highest consumption (3.86 kg/year in Uzbekistan). The wide difference in fish and seafood consumption between countries can be attributed to various factors, including geographical location, income levels and affordability, culinary traditions, and dietary preferences. Tajikistan does not have direct access to the sea. In contrast, Uzbekistan is also landlocked but has better access to imported seafood through trade networks. Countries with coastlines such as Kazakhstan have easier access to fish and seafood. Seafood can be costlier than other protein sources. In countries with lower income levels, people might prioritize more affordable protein options. Educational and awareness factors are also important. Understanding these factors can help explain the wide differences in fish and seafood consumption between countries and inform strategies to promote healthier dietary patterns where needed. Generally, fish and seafood consumption was much below the recommended intake. In contrast to countries with the highest fish consumption, such as the Maldives, Iceland, and Macau (over 70 kg/year/capita), Tajikistan stands among the nations with the lowest fish consumption per capita. It is surpassed only by Ethiopia and Afghanistan, where the consumption figures are 0.53 kg/year/capita and 0.36 kg/year/capita, respectively. The Ministry of Health of Tajikistan recommended 10 kg/year/capita. During the former USSR, per capita annual consumption in Tajikistan was 5–6 kg but due to low domestic fish production and decreased import of fish, the consumption dramatically decreased [66]. A recent study demonstrated that only 3.9% of Tajik women of childbearing age consumed fish [67].

According to a food frequency survey, conducted in Kazakhstan, fish consumption for fishermen was 103 g/day and 49 g/day for non-fisherman [68], corresponding to 721 and 343 g/week, respectively. These values are higher than the 123 g/week later reported for young adults in Kazakhstan (Table 3) [2]. Jia et al. [3] reported the per capita consumption of fish in Kazakhstan of 9.59 kg (184 g/week). In that study, the food consumption data were obtained from the Republic of Kazakhstan Bureau of National Statistics [3], whereas Akhmetova et al. [2] and Hsiao et al. [68] used the food frequency consumption questionnaires. Dietary studies of school-aged children of 9–10 years in Kazakhstan depicted very low fish consumption, especially among the children with obesity [69]. Fish consumption among obese children was 5.8 times lower than the recommended consumption in Kazakhstan.

In the neighboring countries Tajikistan, Kyrgyzstan and Turkmenistan, fish consumption is even lower [70]. In Uzbekistan, residents in Tashkent city and Khorezm consume an average of 1.0 kg and 0.5 kg of fish per year [61]. An assessment of dietary habits of residents in Mary city in Turkmenistan showed the fish consumption of 3.28 kg/year [71]. The current diet in Tajikistan contains only 1% of fish and seafood and mainly relies on bread and vegetables [72].

Consumers with higher education levels tend to eat more fish [71]. Some studies have reported ethnic differences in food consumption [63,73]. Even though the largest ethnic groups in Central Asia are the Uzbek, Kazakh, Tajik, Turkmen, and Kyrgyz, a large proportion of Russian, Ukrainian, and other communities also exist.

**Table 3:** Intake of fish and seafood in Central Asia.

Country	Fish Consumption, kg/year/capita	Notes	Reference
Kazakhstan	3.68	Data from 2021. Estimated for general population. Included total fish and seafood consumption.	[4]
	6.4	Students at universities in all parts of Kazakhstan. Questionnaire data. Included total fish and seafood consumption.	[2]
	27.4	Males. North Central Kazakhstan, Temirtau district, regarded as highly polluted with Hg. Questionnaire data. Included mainly freshwater and demersal fish.	[68]
	14.6	Females. North Central Kazakhstan, Temirtau district, regarded as highly polluted with Hg. Questionnaire data. Included mainly freshwater and demersal fish.	[68]
	37.6	Fishermen. North Central Kazakhstan, Temirtau district, regarded as highly polluted with Hg. Questionnaire data. Included mainly freshwater and demersal fish.	[68]
	17.9	Non-fisherman. North Central Kazakhstan, Temirtau district, regarded as highly polluted with Hg. Questionnaire data. Included mainly freshwater and demersal fish.	[68]
	4.5	Children 9–10 years with normal weight. Almaty. The 24-h recall method.	[69]
	3.1	Children 9–10 years with obesity. Almaty. The 24-h recall method.	[69]
Uzbekistan	3.86	Estimated for general population. Included total fish and seafood consumption.	[4]
	1	General population, Tashkent.	[61]
	0.5	General population, Khorezm.	[61]
Tajikistan	0.63	Estimated for general population. Included total fish and seafood consumption.	[4]
Kyrgyzstan	0.86	Estimated for general population. Included total fish and seafood consumption.	[4]
Turkmenistan	2.51	Estimated for general population. Included total fish and seafood consumption.	[4]
	3.28	General population, Mary City	[71]

## 6. Human Biomonitoring of Toxic Metals in Central Asia

Various studies have been conducted on human populations to determine exposure to toxic compounds. These include both epidemiological studies to determine the health risk associated with exposure to toxic metals and biomonitoring of human populations to assess exposure from the environment. However, only a few studies on biomonitoring of exposure to toxic metals were performed in Central Asia, which were mainly focused on contaminated areas.

Erdinger et al. [74] studied the urine levels of As and Hg in children from two villages in Kazakhstan, namely Aralsk (located in the area close to the Aral Sea, which is regarded as highly polluted) and Akchi (northeastern Kazakhstan). The levels of Hg were numerically but not statistically significantly higher in the children from Aralsk (mean value 0.94 µg/L) compared to Akchi (0.29 µg/L). Both groups of children had Hg levels comparable to or higher than preschool children in China (range 0.03 to 2.63 μg/L) [75], Korea (geometric mean 0.4 µg/L) [76], Italy (range of 0.04–2.18 µg/L) [77], USA (0.7 and 0.9 μg/g creatinine, [78]) and other countries. Those values were below health-based guidance values for the total Hg in urine of 25 µg/L, above which there is an increased risk for adverse health effects [79]. The urine levels of As were 6.4 and 9.6 µg/L in the children from Aralsk and Akchi, respectively.

Children in Pavlodar had Hg concentrations in the hair of 0.44 ± 0.5 mg/kg, which is considered as high and was explained by the activities of metal processing plants and the chemical industry in the Pavlodar region [23]. Moreover, children living in the districts close to the industrial zone had higher Hg concentrations in the hair (up to 0.7 mg/kg).

A cross-sectional study in hospitals in Kazakhstan, Kyrgyzstan, and Uzbekistan detected high concentrations of Pb in the hair of children diagnosed with anemia [80]. Similar results were observed in other countries such as Egypt [81] and India [82]. This might be explained by the fact that the intake of small amounts of Pb can compete with iron absorption and increase the risk of anemia [81]. The same is true for Cd and hair lead and cadmium levels are usually positively correlated [80]. The concentrations of Pb varied from 0.02 to 36.00 µg/g in Kazakhstan and from 1.13 to 27.40 µg/g in Uzbekistan. In Kyrgyzstan, the concentrations of Pb were particularly high and varied from 2.71–50.10 µg/g [80]. Moreover, hemoglobin levels in the children from Kyrgyzstan tended to be lower compared to Kazakhstan and Uzbekistan. Generally, average blood Pb levels have been declining in most countries over the last decade but exposure to Pb during childhood still remains a significant public health problem. Hair Cd and Hg concentrations were highest in Uzbekistan, they were 0.01–1.41 µg/g for Cd and 0.02–2.90 for Hg [80].

## 7. Risk Assessment Based on Intake and Concentrations in Food

In assessing the risk of toxic metal contamination in seafood consumption across Central Asia, several critical factors come to light. The geographical location, pollution sources, water quality, and seafood consumption patterns play pivotal roles in understanding the extent of this environmental challenge. It becomes evident that the effectiveness of existing monitoring programs and regulatory measures is crucial for controlling and mitigating the presence of toxic metals in seafood.

Contamination levels exhibit variations, influenced by both location and season. Notably, major rivers like Amu Darya and Syr Darya show moderate to high pollution levels of toxic metals, including As, Hg, Cd, and Pb. These pollution levels are severe enough to impact aquatic ecosystems, raising significant health concerns for the local population.

Industrial activities significantly contribute to water contamination, exemplified by the impact of the Karbide chemical plant on the river Nura in Kazakhstan. The uncontrolled release of wastewater from such facilities results in elevated levels of mercury, posing health risks for residents in affected areas, such as Temirtau City. In addition to the industrial impact, springs—widely used for various purposes, including drinking water—reveal variable contamination levels. While some springs remain free from Pb, others show elevated Cd levels, potentially posing risks to those relying on these sources for drinking water.

The human health impact is a central concern, with high levels of toxic metals detected in the blood of vulnerable populations, including children and pregnant women, in the lower Amu Darya Basin. This exposure leads to various health issues, such as high infant mortality, low birth weight, growth retardation, acute respiratory diseases, anemia, miscarriage, and other disorders.

Biomonitoring studies further underscore the extent of exposure to toxic metals, such as As and Hg, in children and populations in certain regions. Despite generally falling below health-based guidance values, the presence of these metals in urine and hair suggests ongoing exposure.

Turning attention to the dietary aspects, fish and seafood, integral components of the regional diet, exhibit measurable levels of toxic metals, particularly Hg. Although some studies report levels below permissible concentrations, specific locations, like Lake Balkyldak, reveal high Hg levels in fish. This raises concerns for populations heavily relying on fish as a significant food source. While certain areas show a notable increase in fish and seafood consumption, the overall intake in Central Asia remains below recommended levels. However, in regions with high fish consumption, there is a potential for elevated exposure to toxic metals, especially Hg.

The risk of exposure to toxic metals in fish and seafood in Central Asia emerges as a significant concern. Comprehensive monitoring, regulation, and remediation efforts are imperative to ensure the safety of water sources and food consumption in the region. Public awareness campaigns and the establishment of dietary guidelines play a crucial role in minimizing health risks associated with the consumption.

## 8. Challenges and Future Directions

Addressing toxic metal contamination in Central Asia is an important area of research with several potential future directions. The identification of the major sources of toxic metal contamination in Central Asia, including both present and past industrial activities, mining operations, agricultural practices, and natural sources is urgently needed in order to develop targeted pollution control strategies. The impact of toxic metals on terrestrial and aquatic ecosystems in Central Asia, the potential for biomagnification through the food chain, and the human health implications require further investigations. There is a limited number of epidemiological studies to assess the prevalence of toxic metal-related health issues in populations living in highly contaminated areas. Such studies and long-term monitoring would provide valuable data for trend analysis and policy development. Climate changes should also be considered in future research because climate changes might affect the mobility and bioavailability of toxic metals.

## 9. Conclusions

The accumulation of toxic metals in the environment, either naturally or due to industrial activities, is a serious concern due to their toxicity to humans and other organisms. The level of toxic metals in fish in Central Asia is generally below critical levels and probably does not pose a health risk to the general population considering low fish consumption. Thus, the benefits of fish consumption exceed the possible health effects of exposure to toxic metals. Nowadays, the existing data on toxic metal exposure in Central Asia is insufficient to perform a careful safety evaluation. However, populations in heavily polluted sites might be potentially exposed to high concentrations of toxic metals. It should also be emphasized that toxic metals gain entry into the human body not only via fish and seafood products but also through other foods and water consumption.

## Figures and Tables

**Table 1:** Maximum permissible concentrations of toxic metals in fish according to Eurasian Customs Union [53].

Toxic Metal	Fish Type	Maximum Permissible Concentration
Arsenic, mg/kg	Freshwater fish	1
	Saltwater fish	5
Mercury, mg/kg	Freshwater non-predatory fish	0.3
	Freshwater predatory fish	0.6
	Tuna, swordfish, and beluga—all types of products, including dried products	1
Cadmium, mg/kg	All types of fish products and meat of marine mammals, including dried	0.2
Lead, mg/kg	All types of fish products (except for tuna, swordfish, and beluga) and meat of marine mammals, including dried products	1
	Tuna, swordfish, and beluga—all types of products, including dried products	2

**Table 2:** Occurrence of toxic metals in fish and seafood in Central Asia.

Country	Location	Sample Type	Arsenic, mg/kg	Mercury, mg/kg	Cadmium, mg/kg	Lead, mg/kg	Reference
Kazakhstan	Dam in Bukharzhyrau District, 43°59′53.268″ N 76°35′42.324″ E	Fish: crucian carp (*Carassius carassius*), tench (*Tinca tinca*), perch (*Perca fluviatilis*), roach (*Rutilus rutilus*), carp (*Cyprinus carpio*)	Below LOD	0.0003 ± 0.001	0.0023 ± 0.002	0.0017 ± 0.000	[58]
	Lake Toksumak (Osakarovsky district), 29°16′52.7124″ N 74°20′56.4468″ E	Fish: crucian carp, tench, perch, roach, carp	0.0022 ± 0.002	0.0005 ± 0.000	0.0018 ± 0.001	0.0019 ± 0.001	[58]
	Dam DSU-58 (Nurinsky district), 54°9′19.476″ N 37°34′27.516″ E	Fish: crucian carp, tench, perch, roach, carp	0.0022 ± 0.000	0.0005 ± 0.001	0.0026 ± 0.002	0.0031 ± 0.000	[58]
	Fish nursery of «Livkino» (Uralsk region), 51°22′23.733″ N 51°4′6.25″ E	Fish (no information on species)			0.245 (mean value)	0.25 (mean value)	[59]
	Lake Lastochka (North Kazakhstan)	Fish (no information on species)	0.0132 ± 0.002	0.0026 ± 0.0001	0.0027 ± 0.0002	0.0027 ± 0.0001	[60]
	Lake Kak (North Kazakhstan)	Fish (no information on species)	0.0027 ± 0.0001	0.00206 ± 0.0001	0.0012 ± 0.0001	0.0046 ± 0.0002	[60]
	Lake Tastemirovka (North Kazakhstan)	Fish (no information on species)	0.0130 ± 0.0001	Below LOD	0.0076 ± 0.00001	0.0042 ± 0.0002	[60]
Uzbekistan	Eastern Arnasay Lake System	Common carp (*Cyprinus carpio*)		0.029 (mean value)			[61]
	Lake Tuzkan, 40°39′43″N 67°31′30″ E	Fish: Pike perch (*Sander lucioperca*), aral shemaya (*Alburnus aralensis*), roach (*Rutilus rutilus*), vostrobryushka (*Hemiculter lucidus*)		0.065–0.138 (range)			[61]
	Fish farm Khorezm, 41°20′ N 61°0′ E	Silver carp (*Hypophthalmichthys molitrix*)		0.07 (mean value)			[61]
Tajikistan	Pit Lake, Taboshar, 40°34′12.6″ N 69°38.505′ E	Goldfish (*Carassius auratus*)		0.01 ± 0.01			[54]
	Kairakkum reservoir, 40°16′37.27″ N 69°48′58.02″ E	Pike perch (*Sander lucioperca*)		0.13 (one sample)			[54]
	Kairakkum reservoir	Eurasian carp (not specified)		0.01 ± 0.007			[54]
Kyrgyzstan	Issyk-Kul Lake, 42°29′59.99″ N 77°29′59.99″ E	Issyk-Kul Chebachok (*Leuciscus bergi*)	0.26 ± 0.1	0.062 ± 0.02	0.00091 ± 0.0002	0.022 ± 0.02	[48]
	Issyk-Kul Lake	Pike perch (*Perca schrenkii*)	0.71 ± 0.4	0.066 ± 0.02			[48]
	Issyk-Kul Lake	Rainbow trout (*Oncorhynchus mykiss*)	0.92 ± 0.3	0.026 ± 0.003			[48]
	Chui region, 42°29′59.99″ N 74°29′59.99″ E	Silver carp and common carp		0.078–0.083	0.025–0.03	0.060–0.068	[62]

Data are presented as mean and standard deviations, if not otherwise stated.

## Data Availability

Not applicable.
